# Political orientation of online media sources and reporting of Covid-19 vaccine myocarditis

**DOI:** 10.1371/journal.pone.0296295

**Published:** 2024-01-02

**Authors:** Addison Matsumura, Ria Garg, Muzna Hussain, Martin E. Matsumura

**Affiliations:** 1 Department of Biological Sciences, Oberlin College, Oberlin, OH, United States of America; 2 Internal Medicine Residency Program, Geisinger Health System, Wilkes-Barre, PA, United States of America; 3 The Pearsall Heart Hospital, Geisinger Health System, Wilkes-Barre, PA, United States of America; Damascus University, SYRIAN ARAB REPUBLIC

## Abstract

**Background:**

Political orientation may play a formative role in perceptions of risk associated with COVID-19 vaccination including vaccine myocarditis (CVM). Whether political alignment of news sources plays a role in perception of this risk is unknown.

**Objective:**

We examined the relationship between political orientation of online media sites and aspects of reporting of CVM.

**Methods:**

Media sites were classified as “left” or “right" biased using the Allsides media bias rating report. For each site “COVID vaccine myocarditis” was searched in articles posted May 2021 to December 2022. Each search return was reviewed for the following: 1) Did it contain numerical data regarding CVM risk? 2) Did it report benefits of covid vaccination? 3) Did it mention covid *infection*-related myocarditis? Monthly reports of vaccine-related adverse events were obtained from the Vaccine Adverse Events Reporting System (VAERS).

**Results:**

A total of 487 online reports regarding CVM were reviewed. Comparison of monthly report volumes from left vs. right biased media sources demonstrated significant correlation (r = 0.546, p = 0.013). Additionally monthly reporting of CVM was temporally related to monthly volume of VAERS reporting (r = 0.519, p = 0.023). These data suggest that monthly reporting volumes were driven by availability of information regarding CVM rather than media political alignment. Left biased media sources were significantly more likely to include numerical CVM data vs. right biased sources (76.6% vs. 24.3%, p<0.001) and likewise were more likely to include data supporting benefits of covid vaccination (85.1% vs. 21.7%. p<0.001). In contrast, there was no difference regarding mention of COVID-19 infection-related myocarditis (24.5% vs. 24.3%, p = 0.957).

**Conclusion:**

Political orientation of online news sites was not associated with frequency of CVM reports but was related to report content, most notably whether reports included numerical data regarding CVM risk. These differential reporting characteristics may contribute to the relationship between political orientation and patient conceptualization of risk of CVM.

## Introduction

Within months of the initiation of mass vaccination efforts, reports of COVID-19 vaccine associated myocarditis (CVM) were made to the Center for Disease Control’s Vaccine Adverse Events Reporting System (VAERS) [1–3]. While the incidence of CVM in most analyses is low, it has been shown to be increased in certain demographics, particularly younger males, and at the time of the second of a 2 dose sequence [2, 4]. The possibility of CVM forced a critical assessment vaccination risk and heightened public interest in gaining a better understanding of their individual risk:benefit ratio of vaccination [5].

In a large part due to the polarized political climate in the United States during the COVID-19 pandemic, political orientation became an important factor in shaping individual and collective perceptions of the safety and efficacy of COVID-19 vaccination [6]. While it would be expected that news media outlets would tend to report information in a style that would support or even bolster the views of their readership, it is unknown whether the specific ways in which U.S. news media reported CVM differed by political orientation of either the media outlet or that of their readership. Specifically, it is unknown whether media outlets reported the evolving understanding of CVM with bias toward the political alignment of their readers and whether the timing or content of media reports varied by political bias of individual media outlets. Furthermore, it is unknown whether a degree of mainstream media “misinformation” was truly a factor in the shaping of politically based COVID-19 vaccine opinions.

In the present study we attempted to define differences in the way online news media sites reported CVM based on the political orientation of the site. To define this we assessed both the month-to-month frequency of reporting of CVM by online media sites and whether the volume or content of CVM reports varied with political bias of these sources.

## Methods

The aim of this retrospective review was to determine the volume and specific content elements of online news media sites and the relationship of political orientation of sites to these elements. Online news media sites were identified using the Allsides 2023 media bias rating report [7]. Allsides is a media technology company that uses a nonpartisan panel of reviewers to define the political orientation of over 1200 online news outlets limited to news corporations based in the United States. Allsides defines online media sites as either liberal (“left”) or conservative (“right”) biased based on the panel review of blinded samples from each major media outline site.

Sites were selected from the Allsides “biased left” and “biased right” categories. Sites were included in the data collection if they could be accessed without a fee. For each site, a search was undertaken by a member of the study team using the specific search string “COVID vaccine myocarditis” for articles posted between May 2021 and December 2022. Each identified article was assessed to confirm the primary content was related to vaccine myocarditis and was a news article and not an opinion article. Each confirmed article was reviewed for the following: 1) Did the article include any mention of defined benefits of covid vaccination? 2) Did the article include mention the risk of myocarditis related to COVID-19 viral infection? 3) Did the article contain numerical data to support a discussion of CVM, defined as the presentation of numerical data related to the incidence of CVM, as opposed to qualitative verbiage (i.e., “rare,” “common,” etc.)? These three article characteristics were chosen by the study team as reflecting the balance and evidence basis of the article content. Articles were reviewed and answers to study questions coded in a study database with a designation of “1” for an answer of a “yes” and “0” for an answer of “no” for statistical analysis.

The numerical volume of monthly reports of vaccine-related adverse events were obtained directly from the Vaccine Adverse Events Reporting System [1]. Counts of individual VAERS reports per month were recorded for the months for which media reports were collected. Because of the large volume of reports on the VAERS database for this period, all COVID-19 vaccine side effects were included as opposed to only those addressing CVM.

The word cloud of subjective verbiage pertaining to vaccine myocarditis risk from right-leaning online news media was constructed using an online word cloud creator (Awario, California City CA). Individual descriptive words representing myocarditis risk were identified by the study team member reviewing each article. Word sizes of each word represent the relative frequency of that word’s appearance in news articles.

Correlation between monthly reports of left- vs. right-leaning media was performed using Pearson Product Moment Correlation. For comparison of media reports and VAERS reporting, Spearman Rank Order Correlation was performed with VAERS reports as the independent variable and media reports as the dependent variable. Monthly report volumes were shifted forward 1 month to allow for the lag between VAERS reports and the effect on media coverage (for example, May 2021 VAERS report volumes were compared to June 2021 media reports). Comparisons of proportions of left- vs. right-leaning sites fulfilling the three article characteristics were performed using chi-square analysis on Sigmastat software (Systat, San Jose CA). For all comparisons, p<0.05 was considered significant.

## Results

**Fig 1:**
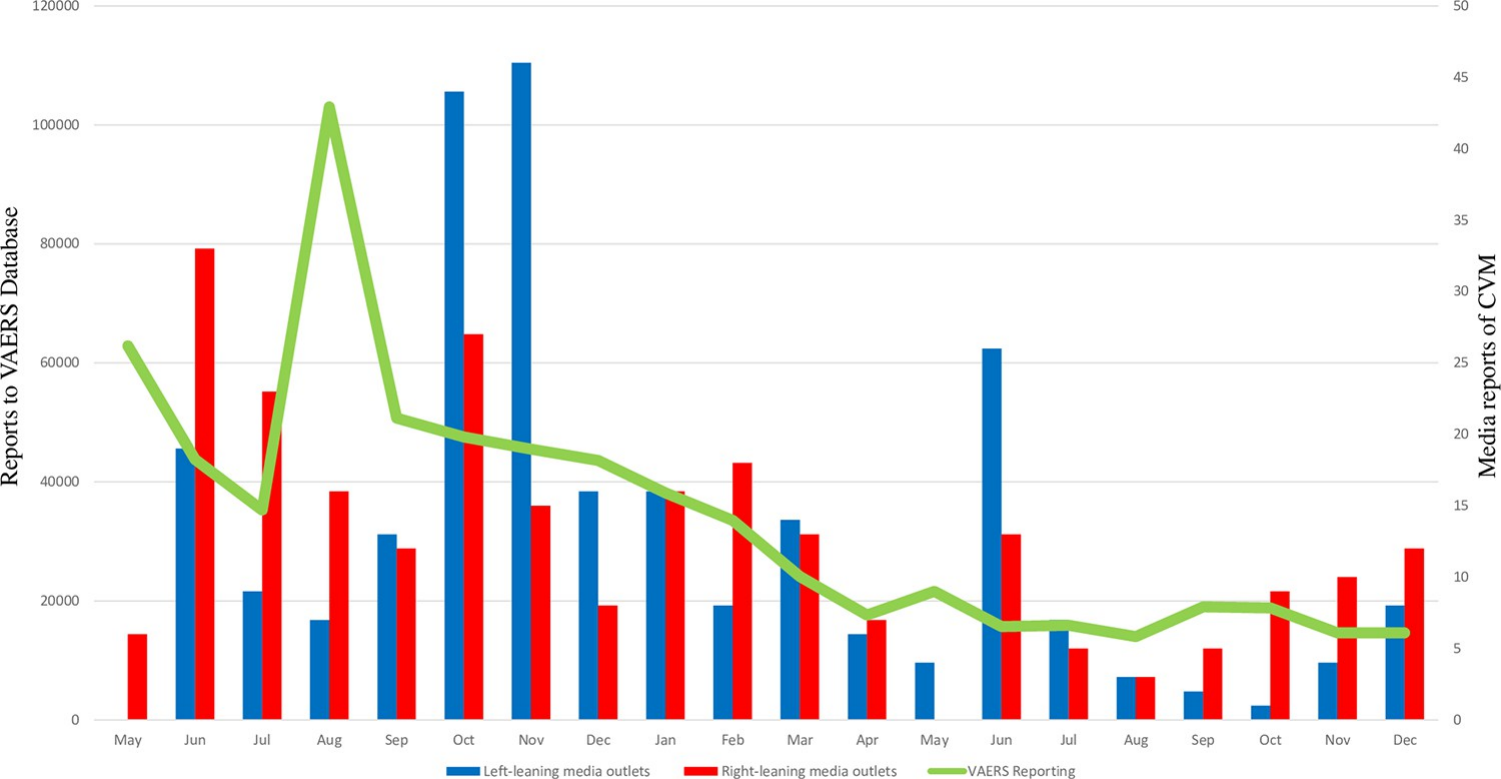
Graph of VAERS reports of COVID-19 vaccine complications and L and R media reports of CVM, by month. Blue bars: monthly reports by left biased media; Red bars: monthly reports by right biased media; Green line: Monthly reports of COVID-19 vaccine adverse events to. VAERS. Correlation of L vs R media r = 0.546, p = 0.013. Correlation of media reports vs VAERS reports r = 0.770, p<0.001. VAERS: vaccine adverse event reporting system.

The monthly volumes of media reports of CVM are depicted in Fig 1. Comparison of total monthly report volumes by online news media with monthly volumes of VAERS reports demonstrated a correlation (r = 0.770, p<0.001) suggesting that total online news reports were likely influenced by VAERS reporting. Additionally, comparison of monthly report volumes between left- and right-leaning media sources demonstrated a strong correlation (r = 0.524, p<0.024), suggesting that monthly reporting volumes through the 2021–2022 time frame were not driven by media political alignment. The relationship of peaks in media reports and monthly volume of VAERS reports suggest that there was interplay between the 2, i.e., the increase in VAERS reports led to increased media coverage and/or increased media reports led to an increase in VAERS activity.

Media sources were compared with regard to specific aspects of content in articles on CVM (Table 1). Left-biased media sources were significantly more likely to include either numerical data or hyperlinks to source material on CVM vs. right-biased sources (76.2% vs. 24.5%, p<0.001) and likewise were more likely to include mention of benefits of covid vaccination in the report of myocarditis (85.1% vs. 21.7%. p<0.001). In contrast, there was no difference between sources regarding mention of COVID-19 virus infection-related myocarditis (24.5% vs. 24.3%, p = 0.957).

**Table 1:** Comparison of percentage of L vs R-leaning media reports of CVM containing specific details/elements.

	Left-leaning media (%)	Right-leaning media (%)	p
	n = 261	n = 226	
Includes numerical data in article	200 (76.6)	55 (24.3)	<0.001
Mentions covid vaccine benefits in article	222 (85.1)	49 (21.7)	<0.001
Mentions covid infectious myocarditis	64 (24.5)	55 (24.3)	0.957

**Fig 2:**
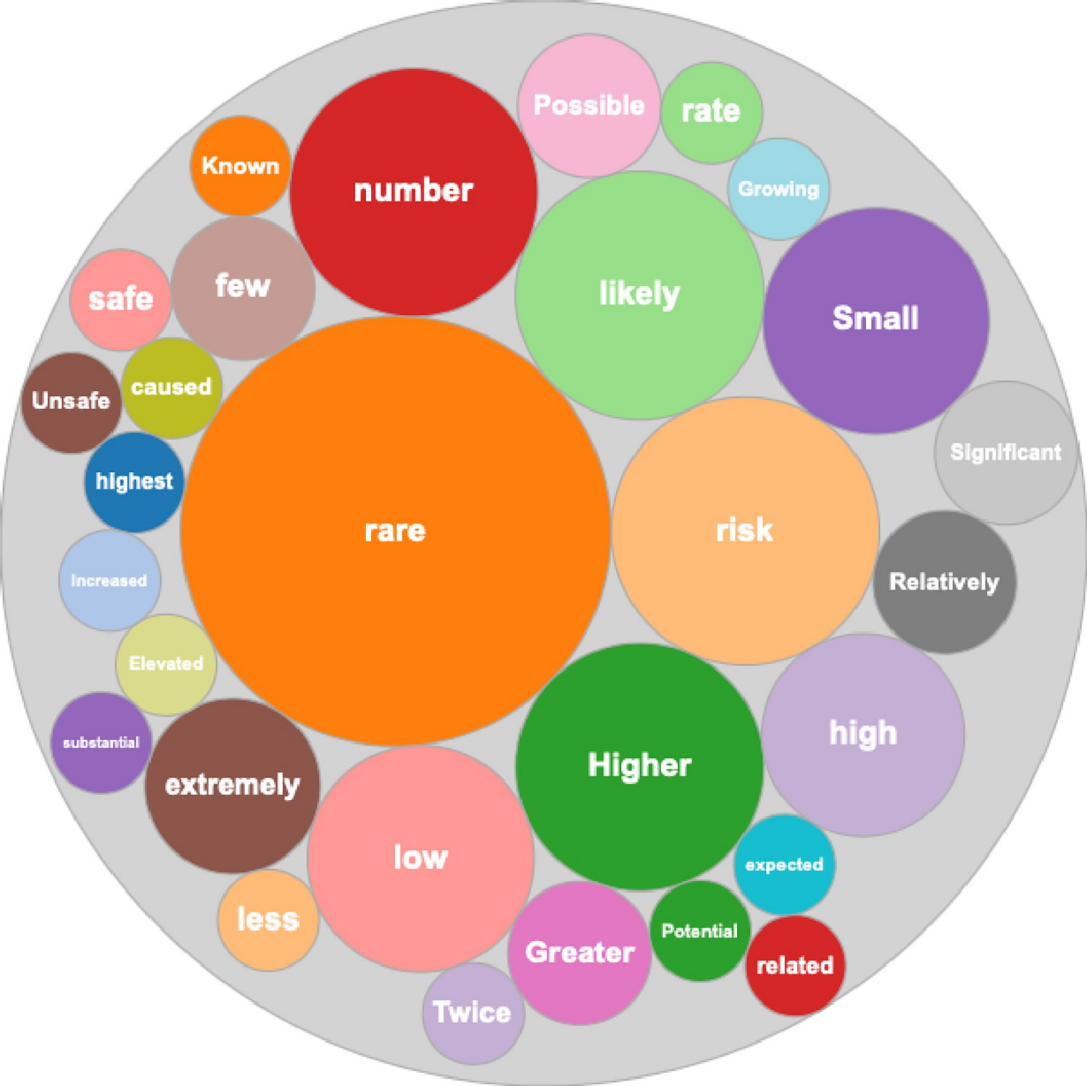
Word cloud representation of qualitative verbiage for risk of CVM. Size of circles is representative of frequency of specific words, either alone or within a phrase.

A word cloud constructed from verbiage representing CVM risk from right-biased news pieces is shown in Fig 2. The size of each bubble represents the frequency of use of each word either alone or in a representative phrase. The most frequently used word either alone or in a phrase was “rare,” and a total of 28 unique words were identified as being used to qualitatively represent CVM risk. Notably, a wide variety of words and phrases were used to represent this risk, highlighting the lack of uniformity among verbiage used to convey a specific risk among media outlets.

## Discussion

In the present study we examined the frequency and presentation characteristics of online news media coverage of CVM from May 2021-December 2022, focusing on difference in coverage between right- and left-leaning media sites. We found that media coverage frequency across political orientation was similar and correlated to the frequency of public reports of CVM to VAERS. While coverage volumes by political orientation were similar, we found significant differences in reports content: specifically, left-leaning sources were significantly more likely to include mention of vaccine benefits and use numerical representation of risk by referring to source data of CVM risk.

Media coverage has had a significant impact on public opinion regarding the COVID-19 vaccine. Prior reports demonstrated a significant impact of media coverage on both willingness to undergo vaccination [8–10] and likelihood to report side effects following vaccine administration [11, 12]. Therefore, any differences in reporting of vaccine adverse events by media source political bias may influence the opinion among readers of vaccine benefits and risks across political ideologies. In the present study we examined how the political bias of media outlets related to the temporal volume and specific content of reports relating CVM. We found that the frequency of reporting CVM during the 2021–2022 Pandemic period correlated with reports of vaccine adverse events to VAERS but that these frequencies did not significantly differ by political bias of the reporting media source. In contrast, we found a significant difference in report content related to media source. Specifically, we found key differences in the representation of CVM risk reporting related to the presence or absence of numerical data related to CVM risk. This difference may have a direct effect on corresponding readers’ impression of risk of this complication, a concept which is supported by prior studies. Rosen et al examined the relationship between numerical versus verbal representations of vaccine myocarditis risk resultant perception of that risk [13]. Those subjects who were presented the verbal descriptor of this risk as “rare” perceived the incidence of myocarditis as significantly higher than those who were presented the numerical probability of 0.001205%. Andreadis et al performed a systematic review of verbal representation in the medical literature and found that numerical interpretation of verbal terms was highly variable among patients; for instance, the term “rare” was interpreted as representing a probability ranging from 7 to 21% [14]. Taken together, these data support a media-based contribution to politically aligned differences in perception of CVM risk and contribute to the stark difference in COVID-19 vaccine confidence between patients of differing political alignment.

The present study has some clear limitations which should be noted. While the finding of a relationship between news media site political orientation and presentation of quantitative numerical data is intriquing, our study provides no direct evidence regarding the effect of this disparity on the formation of an opinion regarding CVM by politically polarized readerships. Furthermore, it is important to point out that omission of numerical or source data is not necessarily indicative or representative of “misinformation” regarding CVM. A lack of quantitative representation of CVM risk should not be considered to be less accurate or scientifically sound reporting. To the contrary, these differences are likely largely reflective of a media outlet’s understanding of its readership’s preferences regarding specific content and style.

In conclusion, our study points out specific differences in media reporting of CVM across the political spectrum. While the factors that shape political opinions are multifactorial and complex, the subtle differences in media coverage of controversial healthcare issues such as CVM could potentially play some role in shaping politically oriented opinions regarding these issues. Knowledge of these differences may help readers and healthcare reporters in frameworking the relationship between medical information sources and public interpretation of such complex and politically-charged topics as complications related to the COVID-19 vaccine.
